# Alcohol Consumption and Behavioral Consequences in Romanian Medical University Students

**DOI:** 10.3390/ijerph18147531

**Published:** 2021-07-15

**Authors:** Bogdana Adriana Nasui, Monica Popa, Anca Dana Buzoianu, Anca Lucia Pop, Valentin Nicolae Varlas, Sebastian Mihai Armean, Codruta Alina Popescu

**Affiliations:** 1 Department of Community Health, “Iuliu Hațieganu” University of Medicine and Pharmacy, 6 Louis Pasteur Street, 400349 Cluj-Napoca, Romania; adriana.nasui@umfcluj.ro (B.A.N.); monica.popa@umfcluj.ro (M.P.); 2 Department of Pharmacology, Toxicology and Clinical Toxicology, “Iuliu Hațieganu” University of Medicine and Pharmacy, 6 Louis Pasteur Street, 400349 Cluj-Napoca, Romania; abuzoianu@umfcluj.ro (A.D.B.); Sebastian.Armean@umfcluj.ro (S.M.A.); 3 Department of Clinical Laboratory, Food Safety, “Carol Davila” University of Medicine and Pharmacy, 6 Traian Vuia Street, 020945 Bucharest, Romania; 4 Department of Obstetrics and Gynaecology, Filantropia Clinical Hospital, “Carol Davila” University of Medicine and Pharmacy, 11171 Bucharest, Romania; valentin.varlas@umfcd.ro; 5 Department of Practical Abilities—Human Sciences, “Iuliu Hațieganu” University of Medicine and Pharmacy, 6 Louis Pasteur Street, 400349 Cluj-Napoca, Romania; cpopescu@umfcluj.ro

**Keywords:** alcohol consumption, illicit drugs consumption, smoking, medical students, risky behaviors, physical activity

## Abstract

Alcohol consumption is a major public health problem facing universities. The objectives of the present study were to investigate alcohol consumption and the behaviors associated with it among Romanian university medical students, with particular reference to sex differences, behavioral consequences, and lifestyle patterns. We performed a cross-sectional study on 722 medical students (60.4% females; 39.6% males); the participants filled out a validated questionnaire containing the following items co-occurring with alcohol consumption: smoking, illicit drug use, energy drink consumption, and other behavioral drinking consequences. Physical activity was estimated using the IPAQ questionnaire. We statistically analyzed the interrelation between alcohol consumption and target factors. The present study showed a high percentage of at-risk drinkers among male (15.0%) and female medical students (14.9%) in the studied group. Male students reported higher illicit drug use and physical activity than female students, but the at-risk female drinkers’ group consumed more drugs than the low-risk female drinkers. Both male and female drinkers engaged in other risky behaviors correlated with drinking (e.g., smoking, low academic performance, and driving a car after drinking). Public health policies, strategies, and interventions should be initiated to reduce alcohol consumption and associated behaviors in medical students.

## 1. Introduction

Alcohol consumption is a considerable public health concern and the leading cause of global suffering. Of particular concern are the health issues and social effects of different alcohol consumption patterns among continents and countries. According to the last World Health Organization (WHO) report, about three million people died due to the harmful use of alcohol in 2016 (5.13% of all deaths) [1]; also, elevated alcohol consumption is recorded in Eastern European Countries, including Romania [2].

Worldwide, heavy episodic (binge) drinking is lower among adolescents (15–19 years) than in the total population, but it is the highest at 20–24 years [2]. From this age range, university students deserve particular attention. University students are at risk for substance abuse behaviors because of changes in lifestyle, reduced parental support, and stress [3]. It is well documented that medical students experience high levels of stress and psychological morbidity, such as depression. They are more likely to use substances, such as alcohol or other drugs, to cope with the stress [4,5]. Stress affecting medical students can lead to lower academic success [6]. Studies from the literature have revealed that medical students drink more than their non-medical—college student peers [6,7]. Young adults consume alcohol in moderation mainly for enjoyment and to enhance mood, but students also drink hazardously, e.g., to cope with stressful life events. Motivations to drink and risk factors for binge drinking and alcohol abuse are both internal and external [8]. Internal factors include, amongst others, genetic predisposition, whereas external factors include early life stress [8]. The most common reasons for alcohol consumption among medical students are social engagement and coping motives [7]. In addition, the prevalence of high-risk alcohol use among college women has been rising since the 1990s, such that we now see virtually the same proportion of males and females reporting binge drinking on a typical drinking occasion [9,10]. Data from the literature evidenced that female medical students drank as much as male medical students [7]. By exceeding the weekly limits, women put themselves at increased risk of such long-term effects as liver disease and breast cancer [11].

A comprehensive review of drinking habits in European Universities found a range of studies suggesting that the hazardous levels of alcohol consumption were associated with increased smoking and drug use [12]. Romania is a European country in transition. The prevalence of illicit drug use among the adult population increased steadily over 2004–2016 after entering the European Union. However, it remains low when compared with other European countries [13]. Furthermore, the consumption of psychoactive substances can lead to dependence syndromes—an array of behavioral manifestations and cognitive and physiological phenomena that develop after repeated use [2].

Another growing concern pertains to the harms of mixing alcohol with energy drinks (substance interactions). International epidemiological studies show that drinkers who consume energy drinks are more likely to record a higher breath alcohol concentration than those who do not; they are more likely to report drinking alcohol in higher quantities, engaging in aggressive acts [14], being injured, and presenting symptoms of alcohol dependence [15]. Of more concern are the at-risk behaviors of medical students due to (1) the influence on the quality of their academic preparation and (2) their role as large-scale health and social influencers to the health of their future patients.

Drinking is a predictor of professional failure among medical students—the greater the amount of alcohol, the higher the amount of failure. Conversely, physicians who exercise, have good eating habits, and abstain from smoking and excessive drinking habits can better provide quality healthcare to their patients and keep themselves healthy [7].

Less is known about the prevalence of alcohol consumption among Romanian medical students and about its association with risky behaviors, so our study aimed to investigate the health-risk behaviors—smoking, illicit drug use, and energy drink consumption—associated with alcohol consumption among these students, with particular reference to sex differences. The study’s second aim was to investigate the behavioral consequences of alcohol consumption (physical problems, driving after drinking, problems with authorities, reduced academic performance, and other behavioral problems related to alcohol consumption). The third aim of the study was to investigate healthy behaviors associated with drinking, such as levels of physical activity. Finally, sociodemographical aspects in the study group were also investigated.

## 2. Materials and Methods

### 2.1. Study Participants

The study was performed during the 2018/2019 academic years. Eligible participants were undergraduate university students (18–30) years, both Romanian and foreign-country students, from the Iuliu Hatieganu University of Medicine in Cluj-Napoca. Cluj-Napoca is a university town with 63,000 students—out of 377,000 students in the country—being the second biggest university town in Romania after the capital, Bucharest. Cluj-Napoca University of Medicine and Pharmacy is the second-largest Medical University in Romania, with 7085 students (National Statistics Institute—N.I.S.).

The Ethics Committee of the Cluj Napoca University of Medicine and Pharmacy approved the study (No. 318/26 July 2018).

We used a multistage cluster method sampling to draw a sample of 722 medical university students. In the first stage, we chose Cluj Medical University, deemed among the seven medical universities in Romania. 

**Figure 1:**
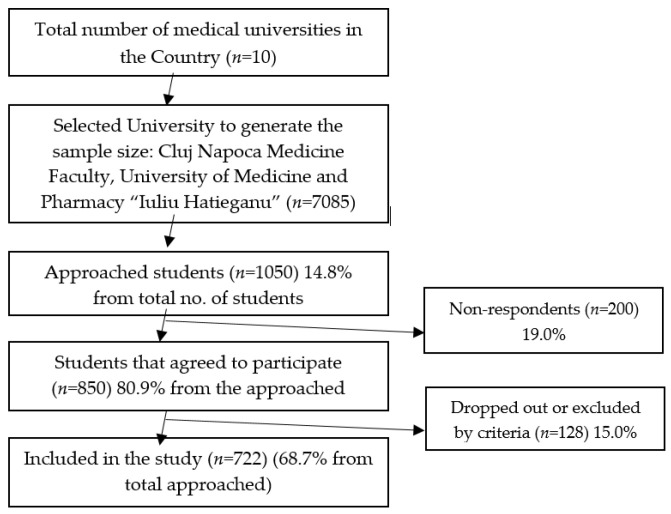
Reporting flow diagram.

According to the Romanian Statistics Institute, we calculated the sample size using Paniott’s formula with an error of 5% based on the total student population; this amounted to 368 participants. Therefore, we calculated a computed minimal size of 659 participants for a 99% confidence level, with a 5% margin of error. Considering a response rate of about 75% (*n* = 790) estimated from a previous study [16,17] and considering a distribution method assuming the non-distracted attention of students at the end of classes, we invited 1050 participants (Figure 1).

Paniott’s formula:

*n* = 1/(Δ2 + 1/N) (1)

D—sample margin of error; N—the total number.

### 2.2. Study Design

A cross-sectional study was done based on the printed questionnaires distributed to the students. After we obtained the Ethics Committee approval and the University’s permission, we contacted student representatives, explained the study and the fact that the questionnaire was anonymous and confidential, and asked them to announce the study to their colleagues. The participation of students in our research was voluntary and not a condition for completing a course. The students did not receive any points counted towards their grades by participating. The University has a mandatory attendance policy in teaching activities, so we sent the printed questionnaire to students upon entrance into the classrooms. Volunteering students took the questionnaire from a desk at the room entrance, brought it home, filled in the questionnaire, and brought it back the next time they came to class. The time taken to complete the questionnaire was, on average, 30 min. By answering the questions, they consented to participate in the study.

### 2.3. Alcohol Consumption

The questionnaires were designed on a previously validated alcohol questionnaire [18]. We calculated the reliability of the Romanian version of the Student Alcohol Questionnaire (SAQ) for the quantity and frequency patterns of the alcohol subscale and certain behaviors—resulting from drinking subscales. Each subscale was subjected to an internal reliability check using the Spearman–Brown spit half technique. We used Cronbach’s alpha as a measure of homogeneity.

The Romanian versions of the six items that assess the quantity and the frequency of drinking beer, wine, and spirits, have a Cronbach’s alpha of 0.80, and the Equal Length Spearman–Brown was 0.81. For the original questionnaire, the Spearman–Brown coefficient was 0.86, and Cronbach’s alpha was 0.84 [18].

The drinking problem subscales included 18 questions. The Equal Length Spearman—Brown coefficient for the Romanian version of these 18 questions was 0.961, and Cronbach’s alpha was 0.925. For the original questionnaire, the Spearman–Brown coefficient was 0.89, and Cronbach’s alpha was 0.82 [18].

The alcohol intake questionnaire assessed the frequency of alcohol use, type, and quantity of beer, wine, and spirits consumed by the student. Students were asked to report the frequency of alcohol units consumed: every day; at least once per week but not every day; at least once a month but less than once a week; more than once a year but less than once a month; once a year or less (Questionnaire available as Supplementary Figure S1).

The quantity of different types of alcoholic beverages was estimated considering the following: more than six drinks; five or six drinks; three or four drinks; one or two drinks; less than one drink.

A standard drink is considered as a glass of wine (150 mL) or beer (330 mL of 5% alcohol) or a shot of a distillate beverage (30–40 mL) [19]. The alcohol frequency questionnaire (AFQ) response categories were assigned constant values to make it possible to calculate units of alcohol per week: (every day = 7.0; at least once a week but not every day = 3.5; at least once a month but less than once a week = 0.5; more than once a year but less than once a month = 0.12; once a year or less or not at all = 0). To compute the drinks of alcohol consumed on a weekly basis, a mean score was calculated by multiplying the quantity by the recorded frequency weight for each beverage type and summing the three scores.

We defined the alcohol unit as the amount of drink generating 10 mL or 8 g of pure ethanol, calculated by the following formula: Units = strength (Alcohol By Volume) × volume (mL) ÷ 1000.

The guidelines of safe alcohol consumption in women are lower than for men, reflecting their increased vulnerability to alcohol-related harm. The standard drink is equivalent to 10 mL or 8 g pure alcohol. In this study, low-risk drinkers were defined as less than 14 drinks per week for women and less than 21 drinks per week for males; overpassing these limits is considered risky drinking.

Through the questionnaire, the study investigated the behavioral consequences of alcohol consumption among medical students: (a) behaviors associated with drinking (smoking, use of illicit drugs, energy drinks consumption), (b) behavioral consequences (physical problems, reduced academic performance, driving after drinking, problems with authorities, and other problems related to drinking).

### 2.4. Assessment of the Physical Activity

To assess physical activity, we used the International Physical Activity Questionnaire (IPAQ) [20]. The physical activity questionnaire estimated physical activities by asking how many days per week one did a vigorous or moderate physical activity. We estimated the duration of physical activity (of at least 10 min) by asking how much time one was involved doing physical activity (hours or minutes per day). Vigorous physical activity refers to activity that requires strenuous physical effort and makes one breathe much harder than usual. Moderate physical activity makes one breathe somewhat harder than normal. Physical activity was coded as low, moderate, and high using the standard IPAQ protocol [21].

### 2.5. Statistical Analyses

We analyzed the data with the IBM Statistical Package for Social Sciences, version 20 (SPSS Inc.^®^, Chicago, IL, USA), and Excel (Microsoft Office^®^ 2010, Albuquerque, NM, USA) using descriptive analyses. The independent variables were sociodemographic data: sex (male, female), age, marital status (unmarried, in a relationship, married, divorced), accommodation (with other friends, at home, rent apartment, university campus, and private campus), religion (yes or no), and ethnicity (Romanian, Hungarian, German, others). The dependent variable was alcohol consumption among medical students. Depending on the level of alcohol consumption, we divided the respondents in low-risk drinkers and at-risk drinkers (as mentioned above).

For comparing the categorical variables, we used the chi-square test. For the variable related to physical activity, the Kolgorov–Smirnov test of normality showed that the data were normally distributed, so we used ANOVA with the Games–Howell posthoc test.

A 0.05 level of confidence (*p* < 0.05) was considered statistically significant.

## 3. Results

### 3.1. Sociodemographics and Patterns of Alcohol Consumption in the Medical Students

**Figure 2:**
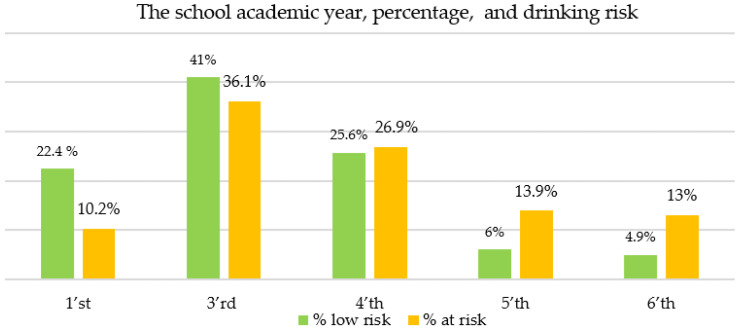
At-risk drinker percentages by school academic year (no data available for the second academic year).

The sample of 722 students consisted of 60.4% (*n* = 436) women and 39.6% men (*n* = 286) due to the specificity of the faculty. The sex distribution of the studied sample was similar to the sex distribution of the students enrolled at the university (according to the official university statistical data, 65% were female, and 35% were male). There were no statistical differences between the sex distribution of our sample and university Romanian medical students (*p* > 0.05, chi square = 3.706). The average age of the students was 22.82 ± 3.06 years among males and 22.01 ± 2.06 among females. The sample included students from urban or rural areas, Romanians of different ethnicities, and international students (Figure 2). The mean age of the sample was 22.34 ± 2.55 years.

The study investigated sociodemographic items related to levels of alcohol consumption. According to the study results, a greater proportion of at-risk drinkers live in rented apartments with other friends or campus accommodations; religion does not influence drinking (Table 1).

Students from the third and fourth years reported higher amounts of alcohol consumption. On the other hand, the results of our study showed a higher percentage of at-risk drinkers compared to low-risk drinkers in the final years of the program (the fifth and sixth academic years) (Figure 2).

**Figure 3:**
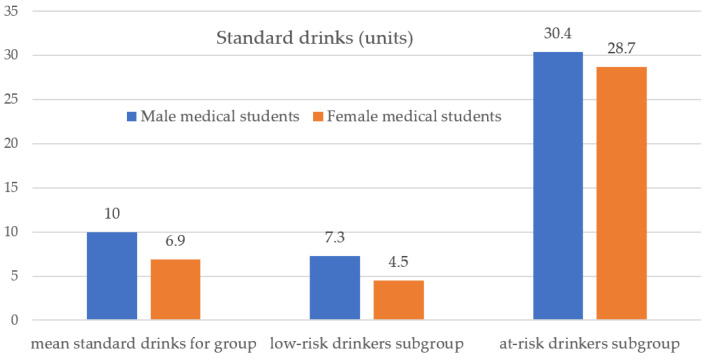
Mean units of drinks per week, by sex (for the entire group and the low-risk and at-risk consumption subgroups).

The study evaluated the mean units of standard drinks (SD) per week. Male students have statistically significantly more SD per week than female students (*p* = 0.04). The present study results evidenced high amounts of ingested alcohol, especially in female medical students (Figure 3).

### 3.2. Behaviors Associated with Drinking

Concerning smoking, the study results showed that both high-risk male and female drinkers smoke in a higher percentage than those who consume moderate units of alcohol. The results evidenced a higher percentage of smokers, both in medical male and female students, even if they are abstainers (Table 2).

Males reported drug use in a higher percentage than females, despite the quantity of alcohol consumed. In addition, the results evidenced that the percentage of female at-risk drinkers who consumed illicit drugs is higher than those who are low-risk drinkers (Table 3).

Energy drinks are consumed by both males and females, regardless of alcohol consumption and whether they are abstainers.

### 3.3. Behavioral Consequences of Alcohol Drinking

The study investigated the behavioral consequences depending on alcohol consumption levels of medical students.

The most frequent consequences of drinking are physical problems. A high percentage of medical students, regardless of the amount they drink, reported a hangover, nausea, or vomiting from drinking. These problems are experienced in a higher percentage by females than males (Table 3).

The study results evidenced that both female and male students experienced problems related to driving after drinking and problems with authorities. These problems were statistically significantly higher among females than males (driving a car after several drinks, *p* = 0.04), but all medical students had trouble with the law because of drinking (Table 4).

Other problems related to medical students who drink are related to academic performance. The present study results revealed that both female and male drinkers missed class or cut class after having several drinks, without any statistical differences between sexes. Unsurprisingly, all at-risk drinker students earned lower grades because of drinking, without statistical differences between males and females (Table 5).

The behavioral consequences of medical students who drink included other potential problems. For example, the study results evidenced that at-risk female students were criticized by someone they were dating because of drinking or were involved in a fight after drinking (Table 6).

### 3.4. Healthy Behavior, Physical Activity Assessment, and Correlations with Alcohol Consumption

The study evidenced that both males and females are engaged in different exercises, but a higher percentage of male students perform vigorous and moderate physical activity (Table 7).

The present study revealed that all medical students meet the amount of physical activity recommended by the WHO (Table 8). However, male students perform more vigorous physical activity than female students (*p* = 0.03), and generally, the amount of physical activity is higher in male students than in female students (*p* = 0.01).

## 4. Discussion

The study’s objective was to estimate the prevalence of alcohol consumption and determine the risky and healthy behaviors associated with excessive alcohol consumption among medical students from Cluj-Napoca. In addition, the study investigated how such consumption is correlated with (a) sociodemographic aspects in the medical school study group (sex and age), (b) behaviors associated with drinking (smoking, illicit drug use, and energy drink consumption), (c) the behavioral consequences of alcohol drinking (physical problems, driving after drinking, problems with authorities, reduced academic performance, and other behavioral problems related to alcohol consumption), and (d) the levels of physical activity and other healthy behaviors.

The present study evidenced a high prevalence of alcohol drinking among medical university students—82.9% among males and 72.8% among female medical students. These results are consistent with previous studies in the same university town on a different sample of students (90% among males vs. 79.8% among females) [22,23]. The findings of our study evidenced a high prevalence of female at-risk drinkers among the student population—similar to other studies taken on other samples of medical students [22]. Alcohol consumption has been noted as the primary public health problem facing universities [24]. The current study evidenced almost no difference in the percentages of male and female at-risk drinkers (15.0% among males vs. 14.9% among women). These results contradict Romania’s social norms and beliefs, whereby female students are assumed to drink less than male students. It is unclear whether this narrowing of the sex gap reflects changing cultural norms or has arisen due to alcohol marketing targeting young women [25].

All drinking studies have shown it to be age-related [26]. The consumption of alcohol increases both in frequency and amount as age increases, as noticed in the present study; first-year students drink less than third-year students. In higher academic years, the percentage of at-risk drinkers is higher than in the first year. These results suggest a potentially increased addictive behavior due to environmental factors during the university years. The results are similar to those of other studies on Romanian students [23] that evidenced frequent alcohol consumption during the academic years. More males drank at all categories of risk than females did and drank more units of alcohol. These results are similar to other studies [27,28].

Associated factors are important to understand the determinants of alcohol consumption and drug use [29]. The present study investigated the sociodemographic profiles of these medical students. Most drinkers, both moderate and at-risk drinkers, live with other friends (in rented apartments or on-campus). These findings support other studies’ findings that evidenced that peer pressure is an associated factor for problematic alcohol use [30]. Family is also a factor that influences harmful alcohol use among adolescents and young adults.

It seems likely that alcohol consumption plays a prominent part in the social life of university students, and that there is an increase during the academic years of medical students, reaching a peak in the third academic year, which is considered the most difficult of medical university, according to Romanian medical school curricula. Students may drink to cope with stress or possibly due to an incorrect perception of alcohol as an entertainment tool, as previous studies have shown [23], a direction that may be further investigated and tackled in public health measures.

Cigarette smoking and drinking commonly co-occur among university students. Results have indicated that medical students who drink alcohol in higher amounts are more frequent smokers [12]. This study found that males smoke in a higher proportion, regardless of the level of alcohol consumed. In addition, at-risk female drinkers are smoking in a greater percentage than moderate female drinkers and at-risk male drinkers. The current study results are consistent with other studies from the literature that showed that students who drink are more likely to smoke [31], and risky drinking was observed among students who smoke [32].

After investigating the illicit drug experiences associated with alcohol consumption among medical students, the present study revealed an increased consumption of drugs with high alcohol consumption. Both males and females subgroups reported increased drug consumption, associated with higher alcohol intake. These results are consistent with studies from other countries that evidenced increased illicit drug consumption among medical students during the latest academic years, both in male and female students [33]. Similar to other studies from the literature, our study evidenced that male students are more often drug consumers than females, regardless of if they are moderate or at-risk drinkers [27,34]. Being male is a predictor of drug use. On the other hand, female at-risk drinkers reported a higher frequency of illicit drug use than moderate alcohol consumers, sustaining other research results [28,35].

Studies from the literature indicated that the consumption of energy drinks and the consumption of alcohol mixed with energy drinks is a relatively common occurrence among students [23,36,37]. Energy drinks contain high levels of caffeine and other ingredients such as taurine or caffeine-containing herbs, such as guarana. Energy drinks can mask the sedative effects of alcohol, such that students underestimate the amount of alcohol they drink, leading to elevated rates of binge drinking, impaired driving, risky sexual behavior, and alcohol dependence when compared with drinking alcohol on its own. Similar to other studies, our study revealed that a high percentage of students consume energy drinks [17,38]. At-risk female medical students ingest a higher amount of energy drinks than other female students, possibly to feel less tired and cope with high study demands.

Consequences of binge drinking include nausea, vomiting, and having a hangover. These results are similar to other studies that evidenced that binge-drinkers are more likely than non-binge-drinkers to experience these drinking consequences, e.g., nausea, vomiting, hangovers, driving a car after drinking, smoking, and illicit drug use [39].

Students who engage in binge drinking are more likely to engage in dangerous behavior than those who do not. In addition, students who drink are involved in other risky behaviors such as driving a car drunk or after several drinks, non-consensual sexual activity, using illicit drugs, and smoking cigarettes or cigars [40]. The results of our study revealed that both males and females reported risky behaviors as a consequence of drinking. However, there are statistical differences among female drinkers regarding risky behaviors. These differences are attributable to females’ sensitivity to alcohol neurotoxicity.

Excessive drinking is associated with several academically related detriments. Researchers have linked excessive drinking to poor classroom performance and test-tasking, missing classes, and falling behind in course work [41]. The present study results showed that drinking among Romanian medical students influenced academic involvement. Both male and female at-risk drinkers show reduced academic performance, e.g., missing or cutting classes. All at-risk drinkers reported receiving a lower grade because of drinking. However, statistical differences were observed between female students. Studies from the literature have shown that both males and females that are binge-drinkers pay less attention in class and are less able to perform specific tasks due to the neurotoxic effect of alcohol on the prefrontal cortex. It is unclear why women have enhanced vulnerability to the neurotoxic effects of alcohol [42].

The results evidenced more statistical differences among female students than male students. In general, women are more prone to alcoholism compared to men based on their body composition. Because women tend to weigh less than men and alcohol is retained in body water, an average woman can consume the same amount of alcohol as an average man but be impacted more [43]. As a result, female drinkers tend to experience adverse effects and develop alcohol dependence [11,44].

On the other hand, more women have been criticized by their partners for excessive alcohol consumption. This consequence may be because alcohol consumption among women is stigmatized.

Several extensive, population-based studies have shown that physically active people are also likely moderate drinkers [45]. Research published in the past decade implies a positive relationship between physical activity and alcohol use, but the results of these studies are inconsistent [17,45,46]. The present study revealed differences between drinkers regarding the level of physical activity. Generally, male medical students performed statistically significantly higher physical activity levels than females, and females walked more than males. Medical students tend to be more informed about the benefits of exercise than other social categories. Both male and female students in the study meet the PA recommendations, which is 30 min of moderate-to-vigorous PA per day, findings consistent with other studies [11]. Regular physical activity is one such lifestyle factor that may help individuals cope with stress and function as an alternative to drinking [27,47]. Marlatt suggested that physical activity may even develop into a “positive addiction,” which positively impacts mood and health [47]. Similar to the present study, previous studies on other medical student groups from Cluj-Napoca evidenced that students are involved in physical activity and meet the WHO recommendations [48].

### Limitations

This study has limitations. The second year of study does not represent medical students due to the lack of data collection possibilities. Moreover, the response rate was lower than we expected since some participants did not retrieve the form or missed classes but gained a minimal number of participants to the study according to the statistical sample size calculation.

Another limitation of the study is the method of a self-administered questionnaire, as the answers can be distorted, but we consider that the voluntary and anonymous participation of the students reduced the possible bias. The inclusion criteria limited the sample to 18–30-year-old medical students, as we intended to exclude the effects of cumulative alcohol behavior and age, the overall number of students in medicine over 30 is meager (according to the official administrative data from the University of Medicine and Pharmacy “Iuliu Hatieganu”). Further studies taking into account older students’ similar alcohol-related behaviors may be needed. A nonresponse bias may influence the results of our study. Since females drink less than males do, our sample comprises 60.4% female, which may generate biases regarding sex differences in alcohol consumption. Further studies are needed to understand the effects of distress on medical students. Higher alcohol consumption was associated with a significantly higher prevalence of engagement in other risk behaviors, particularly substance abuse and sexual behaviors.

Nevertheless, further consolidated studies at a larger scale would show a clearer picture of substance abuse among Romanian medical students. There is a need for improved education regarding alcohol, drugs, and general health in universities. Such education should include all faculties. It remains unclear whether university students’ lifestyles are carried over into later life.

## 5. Conclusions

The present study evidenced a high prevalence of alcohol consumption among Romanian medical students. Furthermore, it showed no differences in the percentages between male and female at-risk drinkers, but there was no clear evidence whether this narrowing of the sex gap reflects changing cultural norms or has arisen due to alcohol marketing targeting young women, enhancing the need for further studies on this topic.

Our study investigated for the first time the correlations between alcohol consumption and health-risk behaviors among medical students in Romania; the results of the study showed that risky behaviors such as smoking, drug use, missing or cutting classes, and driving a car after drinking were associated with high alcohol consumption among medical students.

Meanwhile, public health prevention programs should be implemented among medical students to reduce excessive drinking and harmful behaviors.

## Figures and Tables

**Table 1:** Participant’s sociodemographic and family characteristics.

Variable		Abstainers N (%)	Low Risk N (%)	At-Risk N (%)
Male		49 (17.1)	194 (67.8)	43 (15.0)
Female		97 (22.2	274 (62.8)	65 (14.9)
Marital status	Unmarried	99 (67.8)	290 (62.0)	66 (61.1)
	In a relationship	36 (24.7)	168 (35.90)	36 (33.3)
	Married	6 (4.1)	8 (1.7)	3 (2.8)
	Divorced	5 (3.4)	2 (0.4)	3 (2.8)
Accommodation	With other friends	42 (29.2)	129 (27.9)	31 (29.0)
	Home	22 (15.3)	99 (21.4)	21 (19.6)
	Rent apartment	45 (31.2)	94 (20.3)	22 (20.6)
	University Campus	29 (20.1)	133 (28.7)	29 (27.1)
	Private Campus	6 (4.1)	8 (1.7)	4 (3.7)
Religion	Yes	94 (64.4)	261 (55.9)	56 (51.9)
	No	44 (30.1)	203 (43.1)	52 (48.1)
Ethnicity	Romanian	131 (89.8)	366 (81.8)	82 (75.4)
	Hungarian	4 (2.7)	26 (5.8)	5 (4.6)
	German	4 (2.7)	19 (4.0)	9 (7.7)
	Others	7 (4.8)	39 (8.4)	13 (12.3)

**Table 2:** Behaviors associated with alcohol consumption in the medical school students surveyed.

Variable		Abstainer		Low-Risk Drinkers		At-Risk Drinkers		*p*-Value *
Sex		Male N (%)	Female N (%)	Male N (%)	Female N (%)	Male N (%)	Female N (%)	
Smokers	No	36 (73.5)	70 (72.2)	87 (44.8)	165 (60.2)	14 (32.6)	20 (30.8)	<0.001
	Yes	13 (26.5)	27 (27.8)	107 (55.2)	109 (39.8)	29 (67.4)	45 (69.2)	
Illicit drug experience	Yes	6 (12.2)	4 (4.1)	62 (32.0)	50 (18.2)	21 (48.8)	22 (33.8)	<0.001
	No	43 (87.8)	93 (95.9)	131 (68.)	224 (81.8)	22 (51.2)	43 (66.2)	
Energy drinks	Yes	22 (51.2)	33 (36.7)	100 (51.5)	110 (40.3)	21 (48.8)	32 (49.2)	0.231
	No	21 (48.8)	57 (63.3)	94 (48.5)	163 (59.7)	22 (51.2)	33 (50.8)	

* *p* < 0.05 statistically significant; Chi-Square.

**Table 3:** Physical problems as a consequence of drinking.

Variable—Behavioral Consequences	Female Students		Male Students		*p*-Value *
	Low Risk N (%)	At-Risk N (%)	Low Risk N (%)	At-Risk N (%)	
Had a hangover	217 (88.9)	52 (91.2)	217 (88.9)	30 (90.9)	0.534
Became nauseated and vomited from drinking	222 (90.2)	48 (85.7)	137 (82.0)	30 (78.9)	0.008

* *p* < 0.05 statistically significant; Chi-Square.

**Table 4:** Problems related to driving after drinking and problems with authorities.

Variable—Behavioral Consequences	Female Students		Male Students		*p*-Value *
	Low Risk N (%)	At-Risk N (%)	Low Risk N (%)	At-Risk N (%)	
Driving a car after having several drinks	206 (96.7)	37 (88.1)	122 (92.0)	17 (81.0)	0.044
Driving a car drunk (Driving While Intoxicated)	208 (97.7)	36 (94.7)	122 (93.8)	19 (95.0)	0.094
Getting stopped by police while driving drunk	205 (99.0)	35 (97.2)	122 (96.1)	20 (95.2)	0.075
Experiencing trouble with the law because of drinking	205 (99.5)	206 (100.0)	123 (99.2)	20 (100.0)	0.609

* *p* < 0.05 statistically significant; Chi-Square.

**Table 5:** Problems related to academic performance related to alcohol consumption.

Variable—Behavioral Consequences	Female		Men		*p*-Value *
	Low Risk N (%)	At-Risk N (%)	Low Risk N (%)	At-Risk N (%)	
Coming to class after having several drinks	205 (93.2)	39 (88.6)	131 (94.9)	21 (84.0)	0.454
Missing a class after having several drinks	221 (96.9)	41 (82.0)	145 (94.9)	28 (84.0)	0.363
“Cutting a class” after having several drinks	224 (99.6)	44 (91.7)	146 (97.3)	27 (93.1)	0.235
Earning a lower grade because of drinking	200 (100)	200 (100)	125 (99.2)	20 (100.0)	0.379

* *p* < 0.05 statistically significant; Chi-Square.

**Table 6:** Other problems related to drinking.

Variable—Behavioral Consequences	Female Medical Students		Male Medical Students		*p*-Value *
	Low Risk N (%)	At-Risk N (%)	Low Risk N (%)	At-Risk N (%)	
“Being criticized by someone you were dating because of your drinking.”	203 (99.0)	32 (91.4)	120 (94.5)	19 (95.0)	0.070
“Becoming involved in a fight after drinking”	206 (100.0)	35 (94.6)	120 (91.6)	19 (82.6)	<0.001
Damaging property, causing a false fire alarm, or other behavior after drinking	207 (96.3)	37 (100.0)	126 (91.3)	21 (91.3)	0.014
Participating in a drinking game	224 (95.3)	49 (98.0)	149 (94.3)	28 (100.00)	0.457
Had sex while intoxicated or forcing someone or being forced to have sex	205 (98.6)	36 (94.7)	126 (98.4)	21 (100.0)	0.473

* *p* < 0.05 statistically significant, Chi-Square.

**Table 7:** Physical activity associated with alcohol consumption in medical students.

Variable	Low-Risk Drinkers		At-Risk Drinkers		*p*-Value *
	Male N (%)	Female N (%)	Male N (%)	Female N (%)	
Vigorous PA	135 (71.1)	155 (55.8)	34 (79.0)	41 (63.0)	0.7
Moderate PA	121 (62.3)	152 (55.1)	34 (79.0)	37 (53.8)	0.007
Walking	186 (94.8)	264 (97.4)	42 (97.6)	64 (98.4)	0.9

* *p* < 0.05 statistically significant; ANOVA.

**Table 8:** Levels of physical activity in students and relations to alcohol consumption patterns.

Variable	Abstainers		Low-Risk Drinkers		At-Risk Drinkers		*p*-Value *
Sex	Male	Female	Male	Female	Male	Female	
Vigorous PA (min/week)	285.8	203.9	278.0	233.9	264.7	237.3	0.031
Moderate PA (min/week)	250.0	224.8	268.3	251.8	232.6	187.1	0.442
Walking (min/week)	513.9	589.6	366.1	502.2	593.7	507.6	0.763
Total PA (min/week)	1188.1	1059.8	1134.5	946.1	1131.0	1051.4	0.015

* *p* < 0.05 statistically significant; ANOVA.

## Data Availability

Data available upon request due to ethical and privacy restrictions.

## Supplementary Materials

The following are available online at https://www.mdpi.com/article/10.3390/ijerph18147531/s1. Figure S1: Questionnaire.

## Abbreviations

PA	Physical activity
CI	Confidence Interval
NSI	National Statistics Institute
ESPAD	European School Survey Project on Alcohol and Other Drugs
SPSS	Statistical Package for Social Sciences
WHO	World Health Organization
