# Supplementary material for: Alcohol Consumption and Behavioral Consequences in Romanian Medical University Students
Source: Int J Environ Res Public Health. 2021 Jul 15;18(14):7531. doi: 10.3390/ijerph18147531 (PMC8304642; doi:10.3390/ijerph18147531)
Supplement: Supplementary file 1 [file ijerph-18-07531-s001.zip › ijerph-1261017-SI.pdf]

Questionnaire

1. **Sex** : 1. male 2. female

2. **Age…………….years**

3. **Year of study**: …………….

4. Please think of a typical week during the academic year. Approximate how many hours you spend to prepare for classes and examinations during the week (reading, studying at the library, reports, sought further information) .... hours / week

5. Grades in the previous semester:.................

6.**Ethnicity** : 1. Romanian 2. Hungarian 3. German 4. others (write)...........

7.**Marital status** : 1. single 2. in a relationship 3. married 4. divorced

8. Religion? : Practicant: 1. yes 2. no

9. Accommodation: You live: 1. at home with parents 2. rent apartment alone 3. rent apartment with other friends 4. university campus 5. private campus

10. Do you drink alcohol: 1. yes 2. No

11. What beverage do you usually drink? 1. Wine 2. beer 3. spirits (liquors)

12. How often, on the average, do you usually have a **beer**?:
    1. every day
    2. at least once a week but not every day
    3. at least once a month but less than once a week
    4. more than once a year but less than once a month
    5. once a year or less

13. When you drink **beer**, how much, on the average, do you usually drink at any one time?
    1. more than one six pack (6 or more cans or tavern glasses)
    2. 5 or 6 cans of beer or tavern glasses
    3. 3 or 4 cans of beer or tavern glasses
    4. 1 or 2 cans of beer or tavern glasses
    5. less than 1 can of beer or tavern glass

14. How often do you usually have **wine**?
    1. every day
    2. at least once a week but not every day
    3. at least once a month but less than once a week
    4. more than once a year but less than once a month
    5. once a year or less

15. When you drink **wine**, how much, on the average, do you usually drink at any one time?

    1. over 6 wine glasses
    2. 5 or 6 wine glasses
    3. 3 or 4 wine glasses
    4. 1 or 2 wine glasses
    5. less than 1 glass of wine

16. Next we would like to ask you about **liquors and spirits** (whiskey, gin, vodka, mixed drinks, etc.). How often do you usually have a drink of liquor?

1. every day
2. at least once a week but not every day
3. at least once a month but less than once a week
4. more than once a year but less than once a month
5. once a year or less

17. When you drink **liquor**, how many drinks, on the average, do you usually drink at any one time?

1. over 6 drinks
2. 5 or 6 drinks
3. 3 or 4 drinks
4. 1 or 2 drinks
5. less than 1 drink

18. The following are common problems that other students have reported. If you have never had a drink at all, go to question 19. If you currently drink or have ever drunk in the past, put the number corresponding in the box beside it.

1. Has happened at least once in my life
2. Never happened to me

	Answer (1 or 2)
had a hangover	
gotten nauseated and vomited from drinking	
driven a car after having several drinks	
driven a car when you knew you had too much to drink (Driving While Intoxicated)	
come to class after having several drinks	
missed a class after having several drinks	
"cut a class" after having several drinks	
I was stopped by police driving drunk (after drinking)	
been criticized by someone you were dating because of your drinking	
I had trouble with the law because of drinking (I have done something illegal)	
lost a job because of drinking	
got a lower grade because of drinking	
gotten in trouble with school administration because of behavior resulting from drinking too much	
gotten into a fight/argument after drinking	
thought you might have a problem	
damaged property, pulled a false fire alarm, or other such reckless behavior after drinking.	
participated in a drinking game	
had sex while intoxicated or had been forced or forcing someone to have sex	

19. Do you have a driving license? Yes No
20. Do you had a job? Yes No

21. Do you smoke cigarettes?

Yes

No

22. When you smoke, how many cigarettes do you smoke per occasion? (write in).............no.

23. Have you ever used illicite drugs?

1.Yes 2.No

24. If yes, What drugs have you used?

1.marijuana 2. amphetamines 3. inhalants 4. ethnobotanics 5. others

25. Do you drink energy drinks? 1. Yes 2. No

26. Why do you drink energy drinks?

1. I was curious what they taste
2. to feel full of energy
3. to improve my learning performance
4. to mix with alcohol
5. to stay awake for longer periods of time

27. Do you drink coffee ? 1. Yes 2. No

28. How much coffee do you drink per day?............. ml

We will ask questions about physical activity you perform during a week.

Vigorous physical activities refer to activities that take hard physical effort and make you breathe much harder that normal.

29. During the last 7 days, on how many days did you do **vigorous physical activities** like heavy lifting, digging, aerobics, or fast bicycling,?

Think about only those physical activities that you did for at least 10 minutes at a time**.**

_____**days per week**

none

☐

30. How much time in total did you usually spend on one of those days doing vigorous physical activities?

_____**hours per day**

_____ **minutes per day**

☐ none

Moderate activities refer to activities that take moderate physical effort and make you breathe somewhat harder that normal..

31. Again, think only about those physical activities that you did for at least 10 minutes at a time. During the last 7 days, on how many days did you do **moderate physical activities** like carrying light loads, bicycling at a regular pace, or doubles tennis? Do not include walking

_____ **days per week**

☐ none

32. How much time in total did you usually spend on one of those days doing moderate physical activities?

_____ **hours per day**

_____ **minutes per day**

☐ none

33. During the last 7 days, on how many days did you **walk** for at least 10 minutes at a time? This includes walking at work and at home, walking to travel from place to place, and any other walking that you did solely for recreation, sport, exercise or leisure.

_____**days per week**

☐ none

34. How much time in total did you usually spend walking on one of those days ?

_____**hours per day**

_____ **minutes per day**

☐ none

The last question is about the time you spent **sitting** on weekdays while at work, at home, while doing course work and during leisure time. This includes time spent sitting at a desk, visiting friends, reading traveling on a bus or sitting or lying down to watch television

35. During the last 7 days, how much time in total did you usually spend sitting on a week day?

_____ **hours per day**

_____ **minutes per day**

☐ none

THANK YOU for participating!!
